# Clear Cell Adenocarcinoma of the Renal Pelvis in a Male Patient

**DOI:** 10.1155/2013/494912

**Published:** 2013-12-23

**Authors:** Sarawut Kongkarnka, Pruit Kitirattakarn, Hironori Katayama, Surapan Khunamornpong

**Affiliations:** ^1^Department of Pathology, Faculty of Medicine, Chiang Mai University, Chiang Mai 50200, Thailand; ^2^Department of Surgery, Faculty of Medicine, Chiang Mai University, Chiang Mai 50200, Thailand; ^3^Department of Pathology, Nippon Medical School, Tama-Nagayama Hospital, Tokyo 206-8512, Japan

## Abstract

Carcinoma of the renal pelvis is an uncommon renal neoplasm. Clear cell adenocarcinoma in the urinary tract is rare and has a histomorphology resembling that of the female genital tract. We herein present a case of clear cell adenocarcinoma of the renal pelvis, which is the first example in a male patient to our knowledge. A 54-year-old man presented with right flank pain. The tumor was associated with renal stones and hydronephrosis and invaded into the peripelvic fat tissue with regional lymph node metastasis. The patient died of metastatic disease six months postoperatively. Histologically, the tumor showed complex papillary architecture lined with clear and hobnail cells. Clear cell adenocarcinoma of the renal pelvis may pose a diagnostic challenge on histological grounds, particularly in the distinction from renal cell carcinoma. The immunohistochemical stains could help confirm the diagnosis. Due to its rarity, an effective treatment regimen remains to be determined.

## 1. Introduction

Carcinoma of the renal pelvis accounts for approximately 7% of all renal neoplasms [1]. The majority of cases of renal pelvis carcinoma had conventional urothelial (transitional cell) histology. Adenocarcinoma is a rare type of carcinoma in the renal pelvis, accounting for only 1% of the cases [1].

Clear cell adenocarcinoma (CCA) is a rare histologic subtype of adenocarcinoma in the urinary tract. The tumor has histomorphological features resembling CCA of the female genital tract (or Müllerian origin) [2–4]. To our knowledge, approximately 90 cases of CCA in the urinary bladder and 70 cases in the urethra have been reported [2–6]. However, CCA arising in the upper urinary tract is exceptionally rare [1, 7].

We described a case of CCA of renal pelvis, which, to our knowledge, is the first example in a male patient. CCA in this region may pose a diagnostic challenge on histological grounds, particularly in the distinction from renal cell carcinoma (RCC).

## 2. Case Report

A 54-year-old man presented with right flank pain for eight months. The physical examination detected a palpable mass in the right upper abdominal quadrant. The computerized tomographic scan revealed an enlarged right kidney up to 20 × 15 cm with hydronephrosis and nodular thickening of the pelvic lining, accompanied with enlargement of the hilar lymph nodes. The findings were suggestive of urothelial carcinoma with lymph node metastasis. Multiple renal stones were also present. No clinical evidence or a previous history of any other neoplastic lesion was identified. There was no previous history of any hormonal treatment. The patient was referred to our institution for further surgical management.

Intraoperatively, nephroureterectomy was performed, but there were residual matted lymph nodes extending from the renal hilum with aortic encasement that could not be removed. On gross examination, the cystically dilated kidney showed multiple papillary and solid nodules of pale tan tissue, up to 2.5 cm in diameter, involving almost the entire pelvicalyceal surface with thin residual renal parenchyma. The ureter was free of involvement.

**Figure 1:**
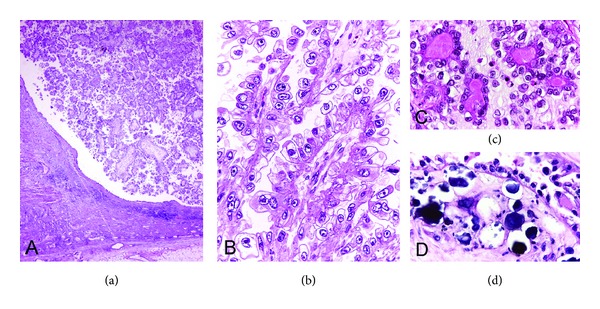
Clear cell adenocarcinoma. (a) Exophytic papillary growth of adenocarcinoma in dilated pelvicalyceal system with adjacent atrophic renal parenchyma (lower left). (b) The papillary structures are lined with hobnail cells with large round nuclei and clear cytoplasm. (c) Deposits of hyaline material in the stroma of the papillae. (d) Psammoma bodies are focally present.

**Figure 2:**
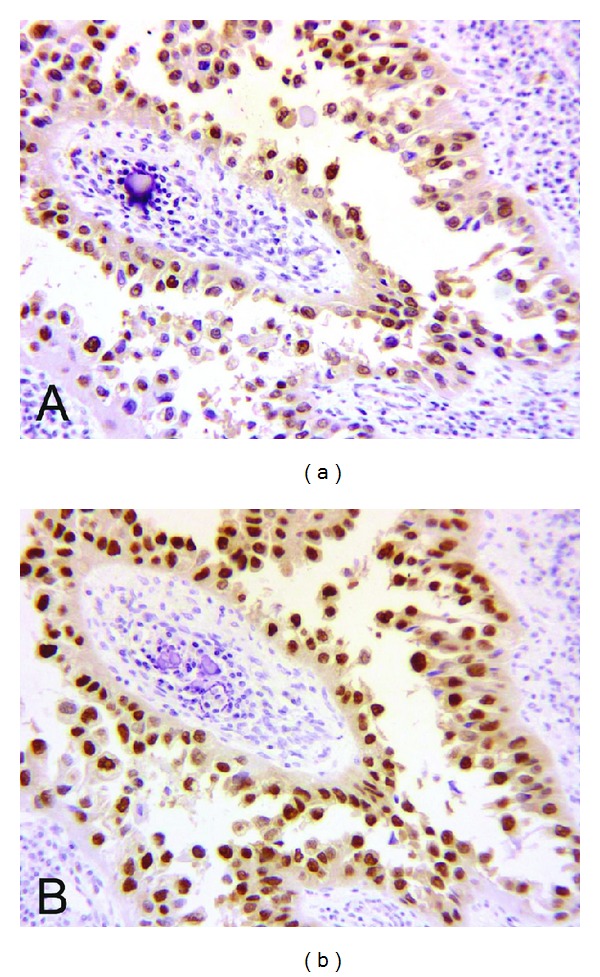
Immunohistochemical stains in clear cell adenocarcinoma. Diffuse positive nuclear staining of hepatocyte nuclear factor-1 beta (a) and PAX8 (b).

Histologically, the tumor was composed of complex papillary structures lined with neoplastic cells with frequent hobnail appearance, clear cytoplasm, and large round nuclei containing distinct nucleoli (Figure 1). The papillary cores contained a variable amount of stroma with occasional deposits of hyaline basement membrane-like material. Concentric and laminated calcifications or psammoma bodies were focally observed. The tumor invaded into the atrophic renal parenchyma and the peripelvic fat tissue. The pelvicalyceal surface was completely lined with clear cells and hobnail cells. No invasive or in situ component of conventional urothelial carcinoma or adenocarcinoma of non-CCA type was identified after a total submission of the renal pelvis for histologic examination in 32 sections. No benign Müllerian-type epithelium was seen. The neoplastic cells showed positive immunoreaction for cytokeratin (CK) 7, hepatocyte nuclear factor-1 beta (HNF), and PAX8, with focal expression of CA-125 (Figure 2). The immunohistochemical stains for RCC marker antigen, CD10, uroplakin III, p63, and CK20 were negative.

Postoperatively, adjuvant chemotherapy was given, composed of vinblastine, Adriamycin, and cisplatin. Multiple pulmonary metastases were detected after three cycles of chemotherapy. He died of the disease six months after surgery.

## 3. Discussion

CCA of Müllerian origin in the female genital tract has a characteristic histologic appearance with papillary and/or tubulocystic pattern and the presence of clear cells and hobnail cells. The occurrence of CCA in the upper urinary system (renal pelvis or ureter) is very unusual. To our knowledge, only one documented case of CCA in this region has been reported [7]. This patient was a 73-year-old woman presenting with left flank pain and elevation of serum CA125 and CA19-9. The tumor mainly involved the upper ureter with extension to the renal pelvis and was associated with the presence of a ureteric stone. The patient did not receive adjuvant chemotherapy and died of metastatic disease five months after the diagnosis [7]. The earlier reports of adenocarcinoma of the renal pelvis or ureter did not specify the histologic diagnosis as CCA or provide the characteristic morphological descriptions or illustrations of CCA [7–9]. Although the tumors in these reports showed CA-125 immunoreactivity, this was not specific for CCA [4].

The histogenetic origin of CCA in the urinary tract is controversial. It has been proposed that the tumor may originate from a remnant or derivative of mesonephric duct or Müllerian duct, nephrogenic adenoma, or urothelium [4–6, 10]. Despite the distinctive morphology that is similar to or indistinguishable from CCA of Müllerian origin and the strong preponderance of female patients [4], the recent investigations using immunohistochemical stains and molecular genetic studies supported the urothelial origin of this tumor [5]. Such findings are in keeping with the presence of coexisting urothelial carcinoma (either invasive or in situ) in one-half and cystitis glandularis in one-third of cases of CCA in the lower urinary tract and the lack of association with endometriosis or Müllerian derivatives [5, 10].

Histologically, CCA of the renal pelvis may pose a diagnostic challenge in the distinction from clear cell or papillary RCC and the other types of carcinoma of urothelial origin showing clear cell features, which include urothelial carcinoma and adenocarcinoma of non-CCA type [1]. In female patients, a possibility of metastasis from CCA of gynecologic origin should also be considered in the differential diagnosis and should be excluded by the clinical evaluation [10]. Immunohistochemical stains could be helpful in the differential diagnosis of carcinoma with clear cell features in the urinary tract as summarized in Table 1 [5, 6, 10–14]. The distinction between CCA and renal cell carcinoma is of paramount importance because of the difference in therapeutic approaches or types of targeted therapy [11]. CCA of the female genital tract and the urinary tract shows characteristic immunoexpressions of CK7, PAX8, and HNF [5, 10, 12, 15]. However, this immunoprofile overlaps with that of papillary RCC, in addition to the histomorphological similarities between both tumors including the presence of psammoma bodies or hyalinized stroma [10, 11, 13, 14]. An association with renal calculi or hydronephrosis and the negative immunoreaction for RCC marker antigen and CD10 would support the exclusion of RCC [11].

The distinction of CCA from other types of urothelial carcinoma with clear cell features is rather an academic concern because their clinical difference has not been determined due to the rarity of CCA in the renal pelvis. Positive immunoreaction for HNF and PAX8 (or PAX2) helps exclude non-CCA types of urothelial carcinoma [10, 14]. Nephrogenic adenoma, a benign lesion in the urinary system which has a tubulopapillary architecture and focal clear/hobnail cells, may be a differential diagnosis of CCA [6]. The presence of evident nuclear pleomorphism, diffuse nuclear hyperchromasia, and high mitotic rate are the major clues in the diagnosis of CCA [6].

CCA in the female genital tract is well recognized as a high-grade and aggressive tumor with a tendency for resistance to chemotherapy [12]. In recent studies on CCA of the lower urinary tract, the tumor seems to have rather aggressive behavior [5, 6]. The majority (76%) of patients had recurrent or metastatic disease (40%) or died of disease (36%), whereas only 24% of patients were alive with no evidence of disease after treatment [5, 6]. CA-125 may be used as a serologic tumor marker in the clinical followup as the neoplastic cells showed CA-125 immunoexpression, and elevated serum CA-125 level was previously reported [7]. Due to the rarity of CCA in the urinary tract, an effective treatment regimen remains to be determined.

## Figures and Tables

**Table 1:** Summary of immunohistochemical stains in the differential diagnosis of clear cell adenocarcinoma in the urinary tract [5, 6, 10–14].

Immunomarker	CCA	RCC	Urothelial carcinoma
Cytokeratin 7	+	+ or −*	+
PAX8	+	+	−
Hepatocyte nuclear factor-1 beta	+	+	−
RCC marker antigen	−	+	−
CD10	− or focal +	+	−
Uroplakin III	− or focal +	−	+

CCA: clear cell adenocarcinoma; RCC: renal cell carcinoma. *Cytokeratin 7 is positive in papillary RCC but negative in clear cell RCC.
